# Growth condition-dependent differences in methylation imply transiently differentiated DNA methylation states in *Escherichia coli*

**DOI:** 10.1093/g3journal/jkac310

**Published:** 2022-12-01

**Authors:** Georgia L Breckell, Olin K Silander

**Affiliations:** School of Natural and Sciences, Massey University, Auckland 0745, New Zealand; School of Natural and Sciences, Massey University, Auckland 0745, New Zealand

**Keywords:** nanopore, DNA methylation, *E coli*

## Abstract

DNA methylation in bacteria frequently serves as a simple immune system, allowing recognition of DNA from foreign sources, such as phages or selfish genetic elements. However, DNA methylation also affects other cell phenotypes in a heritable manner (i.e. epigenetically). While there are several examples of methylation affecting transcription in an epigenetic manner in highly localized contexts, it is not well-established how frequently methylation serves a more general epigenetic function over larger genomic scales. To address this question, here we use Oxford Nanopore sequencing to profile DNA modification marks in three natural isolates of *Escherichia coli*. We first identify the DNA sequence motifs targeted by the methyltransferases in each strain. We then quantify the frequency of methylation at each of these motifs across the entire genome in different growth conditions. We find that motifs in specific regions of the genome consistently exhibit high or low levels of methylation. Furthermore, we show that there are replicable and consistent differences in methylated regions across different growth conditions. This suggests that during growth, *E. coli* transiently differentiate into distinct methylation states that depend on the growth state, raising the possibility that measuring DNA methylation alone can be used to infer bacterial growth states without additional information such as transcriptome or proteome data. These results show the utility of using Oxford Nanopore sequencing as an economic means to infer DNA methylation status. They also provide new insights into the dynamics of methylation during bacterial growth and provide evidence of differentiated cell states, a transient analog to what is observed in the differentiation of cell types in multicellular organisms.

## Introduction

Cellular phenotypes are determined not only by genetic and environmental factors but also by epigenetic factors (heritable changes to the phenotype which are not caused by changes to the DNA sequence). In bacteria, epigenetic inheritance of phenotypes is known to occur via a range of mechanisms, including transgenerational inheritance of transcription factors or membrane transport proteins (Lambert and Kussell 2014; Kaiser *et al*. 2018), protein aggregates (Govers *et al*. 2018), or by covalent modifications to DNA (Hale *et al*. 1994; Sánchez-Romero and Casadesús 2020). Three types of covalent DNA modifications commonly found in bacteria, all mediated by the addition of methyl groups, are C^5^-methyl-cytosine (5mC), C^6^-methyl-adenine (6mA), and N^4^-methyl-cytosine (4mC) (Sánchez-Romero *et al*. 2015; Blow *et al*. 2016; Beaulaurier *et al*. 2019; Oliveira 2021). Methylation at these sites occurs via the action of DNA methyltransferases (Casadesús and Low 2006; Jablonka and Raz 2009; Heard and Martienssen 2014), which are ubiquitous across bacteria (Oliveira and Fang 2021).

Despite the ubiquity of DNA methylation, how often it serves an epigenetic function in bacteria is not well-established. In many cases, DNA methylation does not lead to different heritable phenotypes and thus does not function as an epigenetic mark, for example, the well-studied case of transiently hemi-methylated DNA at the *dnaA* locus after replication initiation (Skarstad *et al*. 1986; Collier 2009; Waldminghaus and Skarstad 2009). As this hemi-methylation is only transient and not inherited across generations, it is not generally referred to as epigenetic. However, a number of studies have established that DNA methylation does act to regulate cellular processes, including gene expression (Roberts *et al*. 1985; Seong, Han, *et al*. 2021), and this can be heritable with significant downstream phenotypic effects (van der Woude *et al*. 1998; Low *et al*. 2001; Casadesús and Low 2006; Park *et al*. 2019; Sánchez-Romero and Casadesús 2020). Notably, in almost all well-established cases, when DNA methylation functions in an epigenetic manner, it is highly localized (e.g. at the operon level) (Hale *et al*. 1994), or even at single sites (Birkholz *et al*. 2022). One exception to this is differences in genome-wide DNA methylation patterns observed between free-living and terminally differentiated bacteroids of the soil bacterium *Rhizobium leguminosarum* (Afonin *et al*. 2021).

To further probe possible epigenetic functions of DNA methylation in bacteria, here we characterize methylation patterns for three natural isolates of *Escherichia coli* across a wide range of growth conditions. We profile DNA methylation using Oxford Nanopore (ONT) sequencing (Rand *et al*. 2017; Simpson *et al*. 2017) and show that by comparing samples of native methylated genomic DNA to whole-genome amplified DNA it is possible to identify the expected methyltransferase binding motifs. We then use a quantitative sliding-window approach to show that across the genome, methylation levels vary in a predictable fashion and that levels of methylation differ between growth conditions. These data suggest that *E. coli* cells undergo environment-dependent transient differentiation into different methylation states during growth. These changes are not a reflection of cell cycle states, but instead are heritable changes that are gradually lost after growth ends. These results raise the possibility that in bacteria, growth states can be inferred solely by quantifying DNA methylation patterns, and that these patterns correspond to transiently differentiated epigenetic cell states.

## Methods

### Bacterial growth

We grew overnight cultures from single colonies for each natural isolate in 3 ml of liquid LB media at 37°C. We then inoculated 75 ml of the relevant growth media (either LB or M9 minimal media with 0.2% glucose) in a 250-ml Erlenmeyer Flask with 75 µl of overnight culture. We grew these at the relevant temperature (37°C, 25°C, 42°C) until an OD600 between 0.4 and 0.5 was reached, or for 24 or 96 h (for WGA and late stationary phase samples). 5 ml of media was removed into a 15-ml falcon tube and the cells were pelleted by centrifugation at 14,000 rpm for 4 min. We removed the media and spun the cells for an additional 2 min, after which we pipetted off any remaining media. We stored the cell pellets at −20°C until DNA extraction.

### DNA extraction and whole-genome amplification

We extracted DNA using the Promega Wizard DNA extraction kit following the gram negative bacterial extraction protocol. We performed whole-genome amplification (WGA) using the Qiagen RepliG kit according to the manufacturer's protocol. We used a Qubit fluorometer to measure DNA concentration, ensuring that each sample had sufficient DNA for a ligation library prep without further concentrating the sample. We measured DNA purity with a Nanodrop. For all samples, the 260/230 and 280/230 ratios were between 1.5 and 2.3. We stored DNA at −20°C until library preparation and sequencing.

### Library preparation and DNA sequencing

We prepared ONT sequencing libraries for both the WGA and native DNA using either the SQK-LSK109 kit with barcode expansion kit EXP-NBD104 or the SQK-RBK004 kit. For the SQK-LSK109 kit, we followed the manufacturer's protocol with no modifications. We modified the SQK-RBK004 protocol as follows: we eluted the samples off Agencourt Ampure XP beads using TE buffer pre-warmed to 50°C; we performed the elution itself at 50°C; and we increased the incubation time for elution to 10 min.

We performed ONT sequencing on a MinION Mk1B device using R9.4.1 flowcells. We used eight flowcells in total (two with SQK-RBK004 libraries and six with SQK-LSK109 libraries), with 12 samples run per flow cell. One additional flow cell was used to produce an additional 1 Gbp for a single sample that had low coverage. For each sequencing run, we demultiplexed and basecalled using Guppy v4.2.2.

For quantitative analysis of methylation, we subsampled all WGA and native sequencing reads to ensure even coverage across the genome using the following strategy: for each sample, we mapped all reads onto the relevant reference genome and determined the lowest 5th percentile of coverage over all samples, excluding the 96-h sample, which had lower coverage for all strains (see below). For the 96-h samples, we calculated the 5th percentile of coverage only for those samples, rather than across all samples.

We then standardized coverage across the chromosomal contig at this 5th percentile level. We first calculated the mean read length for each dataset. We then divided the genome into 10-Kbp windows and sampled an appropriate number of reads originating within each window such that the read length and the target coverage matched (e.g. if the mean read length was 2 Kbp and the target coverage was 100X, then, we selected 500 reads originating within the 10-Kbp window). We then mapped all reads back onto the genome to confirm that we had reached the coverage targets. If the target coverage was not achieved (for example due to irregularities in the read length distribution), the mean read length was adjusted to represent the mapped reads, and reads were resampled. We then used the ONT-fast5-api to extract the corresponding fast5 reads for each dataset (see https://github.com/GeorgiaBreckell/Methylation).

### Identification of methyltransferases

We previously produced reference-level genomes for each strain (Breckell and Silander 2020) using Prokka (Seemann 2014). We identified methyltransferases by using bwa mem (Li 2013) to map all restriction enzymes and methyltransferase enzymes in the REBASE Gold database (Roberts *et al*. 2010) to each strain. The REBASE Gold database contains only experimentally validated methyltransferase and restriction–modification (RM) systems. We filtered the alignments to include only those genes which aligned for more than 97% of their length.

### DNA modification analyses

#### Detection of modified sites using Nanodisco

We used Nanodisco to detect DNA methylation (Tourancheau *et al*. 2021) with the recommended default settings. We processed fast5 reads from both WGA and native DNA samples separately with the Nanodisco preprocess command before running the Nanodisco difference command to calculate differences in the WGA and native DNA signals at each position. We used the Nanodisco merge command to create a single output file containing the native and WGA coverage for each genomic location, the mean signal difference and *U*- and *t*-test *P*-values reporting the significance of the signal difference at each site.

#### Quantification of methylation at individual sites

The Nanodisco output includes a *P*-value of a two-tailed Mann–Whitney *U* test for each site indicating whether or not that the signal at that site differs between the modified and unmodified samples. However, this *P*-value is not necessarily lowest at the actual point of modification, as the nanopore detects five bases at once, and the methylation can affect the signal in unpredictable ways. For example, many bases that were identified as having signals that differed between native and WGA DNA were not highest at the expected cytosine position within GATC motifs. To ensure we identified methylated motifs, we first identified all motif locations (DCM and DAM) in the genome (CCWGG and GATC, respectively), and then identified the lowest *P*-value out of the focal base and either neighboring base. We used this *P*-value as an indication of whether or not a CCWGG or GATC site was methylated.

To account for false-positive identification of modified sites, we used the *P*-values from above for the DCM and DAM sites located in the first 1 Mbp of the genome. We also identified an equal number of random locations in the first 1 Mbp of the genome, and identified the lowest *P*-value of each random base pair or either neighboring base pair. We performed this analysis only in the first 1 Mbp of the genome to minimize computational effort; it is highly unlikely that this has any effect on the results. This resulted in a set of *P*-values for possibly methylated sites within each target motif, and likely unmethylated random sites. We used the *P*-values from the random sites to establish a null distribution of *P*-values for unmethylated bases. We designated all DAM and DCM sites with *P*-values lower than the 10th percentile of the null distribution as methylated (Supplementary Fig. 1). All other DAM and DCM sites we designated as unmethylated. The precise implementation of this method is available through the github repository indicated above.

#### Correlation in methylation fractions

To calculate correlations in the fraction of methylated sites, we first determined the number of DAM or DCM binding motifs within each 10-Kbp window for each genome. We used this as an estimate for the number of potential DAM or DCM modifications and then calculated the fraction of DAM or DCM sites that we experimentally identified as modified in each window. We calculated the correlation between the fraction of modified sites in each window as a Pearson correlation or a partial correlation accounting for sequencing coverage, as sequencing coverage affects the likelihood that a site will be detected as modified.

#### Genome-wide methylation patterns

We assessed genome-wide methylation patterns by comparing the fraction of known sites vs modified sites in windows across 10-Kbp windows in the genome. We discarded any regions that contained no DAM or DCM sites, as this would result in a division-by-zero problem. For the normalized data presented in Supplementary Fig. 6, we simply divided the fraction of methylated sites in each window by the mean of all windows across the genome.

## Results

### Determination of methylation motifs

We first sought to determine which methyltransferases were present in each of three natural isolates of *E. coli*, denoted here as SC419, SC452, and SC469 (Ishii *et al*. 2006). Mapping all restriction modification enzymes in the REBASE Gold database (‘see *Methods*’), we found the adenine methyltransferase *dam* (which recognizes GATC motifs) and the cytosine methyltransferase *dcm* (which recognizes CCWGG motifs) in all three strains. We also found one of the adenine methyltransferases EcoKII or EcoGVI (originally named YhdJ (Broadbent *et al*. 2007)) in each of the three strains. Both targets the same motif, ATGCAT (Broadbent *et al*. 2007), and are present in most *E. coli* strains (Fang *et al*. 2012; Adzitey *et al*. 2020). We also identified the methyltransferase EcoGIX in strains SC419 and SC469. EcoGIX is an adenine methyltransferase, with a loosely defined motif sequence (Fang *et al*. 2012; Forde *et al*. 2015). Finally, we identified EcoGVII in strain SC469, which is a close homolog of DAM (Fang *et al*. 2012), and recognizes the same target motif.

**Fig. 1.**
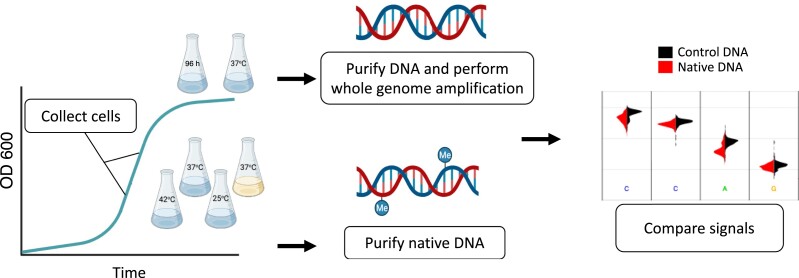
Experimental design for sampling native (possibly modified) and unmodified DNA. To sample native DNA, we grew cultures until exponential phase (for the minimal M9 media, rich LB media, 42°C and 25°C growth conditions); or late stationary phase (for the 96-h growth condition). For whole genome amplification, we isolated DNA from early stationary phase (24 h of growth). After purification of genomic DNA (and whole-genome amplification when necessary), we sequenced the samples using the ONT platform. To infer DNA modifications, we compared the signals from native and WGA DNA using Nanodisco.

To determine whether each of these methyltransferases were active, we used ONT sequencing to identify genomic sites where DNA was modified (Fig. 1). We sequenced native DNA which may contain modified bases, and whole-genome amplified (WGA) DNA which should contain no modifications. We generated at least 50-fold genomic coverage of ONT data from native DNA and at least 100-fold genomic coverage of ONT data from WGA DNA (‘see *Methods*’). Note that these fold-coverage values are mean coverage values over the whole genome. To determine which genomic sites were modified we used a simple statistical approach implemented by Nanodisco (Tourancheau *et al*. 2021). Nanodisco uses the differences in the raw nanopore signals from each sample to assign a *P*-value to every position in the genome using a Mann–Whitney *U* test (‘see *Methods*’).

We selected flanking regions from the 5,000 bases with the lowest *P*-values for input into MEME (Bailey *et al*. 2009) to identify motifs associated with modified bases. However, we found that in almost all cases, MEME identified only the cytosine methyltransferase DCM motif (CCWGG). We hypothesized that this was because methylated DCM motifs generally have smaller *P*-values than other motifs, due to larger signal deviations from unmethylated motifs. Because there are more than 13,000 DCM sites in each genome, the vast majority of the regions with low *P*-values will be DCM sites, even when considering a very large number of sites (e.g. more than 10,000). We found that using a larger number of regions for input into MEME was computationally prohibitive. We thus randomly subsampled 100,000 base pairs (and associated *P*-values) from the genome (representing approximately 2% of the genome). From this subsample, we selected the flanking regions for the 5,000 base pairs with the lowest *P*-values for input into MEME.

For all three strains, MEME identified GATC and CCWGG as significant motifs (Table 1). These are the canonical motifs for the DAM and DCM methyltransferases, respectively, and we had bioinformatically identified both in all three strains. As these matches the DAM and DMC motifs, we assume that they contain C^6^-methyl-adenine (6mA) at the A position and C^5^-methyl-cytosine (5mC) at the second cytosine, respectively. Although we computationally identified the adenine methyltransferases EcoKII and EcoGVI in the three strains, we did not identify their target motif ATGCAT in any strains. We speculate that this is because methylated adenines are more difficult to identify (see above), and because this 6-bp motif is considerably more rare than the four-base pair motifs recognized by DAM and DCM. We also identified methyltransferase activity at two additional motifs, CCGG and GAGCC, in SC419 and SC452, respectively. Although there are no experimentally validated methyltransferases in the REBASE Gold database that are known to target these motifs, there are a number of putative type III RM system methyltransferases that are thought to target these motifs. We mapped the sequences of each of these putative methyltransferases against each genome and identified a single genomic region in SC452 that matched all the putative GAGCC modifying methyltransferases (Table 1). This methyltransferase has a non-palindromic motif, and thus methylates only a single strand (Meisel *et al*. 1992). Surprisingly, we did not identify any CCGG-targeting methyltransferase in the SC419 genome. Finally, for the last two computationally identified methyltransferases, EcoGIX and EcoGVII, we could not confirm their activity. This is not unexpected, as the EcoGIX motif is ambiguous and the EcoGVII motif overlaps with DAM.

**Table 1.** Matches between sequence motifs identified by MEME and REBASE Gold methyltransferases.

Strain	Target motif reported by MEME	Number of motifs identified in 100 Kbp	MEME *P*-value	Inferred REBASE Gold enzyme
SC419	CCWGG	632	3.1e-457	DCM
	GATC	625	4.9e-177	DAM
	CCGG	376	2.3e-259	unknown
SC452	CCWGG	750	2.3e-628	DCM
	GATC	681	3.3e-235	DAM
	GAGCC	111	4.1e-24	M.EcoB0880RFEP*^a^*
SC469	CCWGG	371	1.2e-212	DCM
	GATC	185	1.4e-30	DAM

Each row indicates the top three motifs as reported by MEME.

This is a putative methyltransferase that is not found in the experimentally confirmed REBASE Gold database.

### Quantitative analysis of methylation levels

We next sought to determine whether there was variation in the levels of methylation across the genome, or whether all regions were equally methylated. We focused only on the most commonly methylated motifs in each genome, GATC (containing methylated adenines via DAM) and CCWGG (containing methylated cytosines via DCM). Critically, we found that the likelihood that a site is identified as methylated depended on the coverage of that site (Supplementary Fig. 1). Thus, to increase the likelihood that all sites across the genome had an equal probability of being identified as methylated, we subsampled each of the ONT sequencing datasets to standardize coverage across the genome (‘see *Methods*’).

We then used Nanodisco to compare the native and WGA datasets for all three genomes, and for each known DAM and DCM motif site took the lowest *P*-value from within the 3 bp surrounding each motif (‘see *Methods*, *Quantification of methylation at individual sites*’). These *P*-values should be indicative of the methylation status of a site, as they result from a Mann–Whitney *U* test comparing the signal levels of modified and unmodified DNA. In addition, we hypothesized that sites at which all DNA molecules have a methylated nucleotide would have smaller *P*-values compared to sites at which only a small number of molecules are methylated, and that *P*-values are thus a quantitative indication of methylation status.

**Fig. 2.**
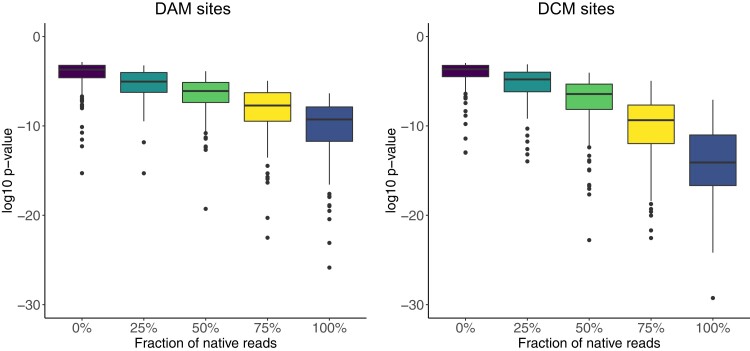
The *P*-values resulting from Mann–Whitney *U* tests for signal deviations at DAM and DCM sites are correlated with the fraction of methylated molecules. We mixed known fractions of WGA reads (unmethylated) and native reads (possibly methylated) in silico and used Nanodisco to determine the *P*-value of a Mann–Whitney *U* test at each position in the genome. We then determined the lowest *P*-value in a 3-bp window surrounding each hypothetically modified base in DAM (GATC) or DCM (GGCC) motif. For both methyltransferases, the sensitivity of the test increases as the fraction of native reads increases, with the DCM *P*-values decreasing to a much larger extent.

To directly test this hypothesis, we subsampled reads from the WGA data (which arises from fully unmethylated reads) to reach 50 × coverage across the genome. We compared this WGA data with mixed native and WGA datasets having 50 × coverage but consisting of 0%, 25%, 50%, 75% or 100% native reads. We then used Nanodisco to infer methylation status for all positions in the genome in these datasets with different ratios of WGA and native reads. We found a clear negative relationship between the fraction of native reads in the dataset and the associated *P*-values for each position (Fig. 2): as the fraction of native (possibly methylated) reads in the dataset increased, the *P*-values decreased. This indicates that the *P*-values returned by Nanodisco are correlated with the fraction of methylated molecules at a site, and may provide quantitative insight into the fraction of molecules that are methylated at any DAM or DCM position in the genome.

We then implemented a simple binary classification of DAM and DCM sites in the native DNA as being methylated or unmethylated (or less methylated) using a *P*-value cutoff (Supplementary Figs. 2 and 3). We placed this cutoff such that 10% of non-methylated sites were inferred as being methylated, analogous to implementing a false discovery rate of 0.1 (‘see *Methods*’; Supplementary Figs. 4 and 5). Although it would also be possible to implement a generative model specifying the fraction of molecules that are methylated at any one location in the genome, without a ground truth set of data for both unmethylated and methylated molecules, this is difficult. Thus, we used this simple binary classification. Importantly, this division into methylated and unmethylated status for each site does not indicate definitively that a site is methylated or unmethylated. Rather, the division establishes that specific sites are more or less methylated (Fig. 2). We then used this classification of sites as methylated or unmethylated to test whether there were consistent differences in methylation rates across the genome or across growth conditions.

### Identification of local and global methylation patterns

To test for differences in methylation across growth conditions, for each strain we isolated DNA from cultures grown to exponential phase in five different conditions: two replicate cultures grown at 37°C in minimal media (M9 glucose), one grown at 37°C in LB broth (rich media), one grown at 25°C in minimal media (cold stress), one grown at 42°C in minimal media (heat stress), and one after 96 h of growth in minimal media (late stationary phase). For each of these growth conditions, we performed the same analyses outlined above to determine whether DAM and DCM sites were classified as methylated or unmethylated.

**Fig. 3.**
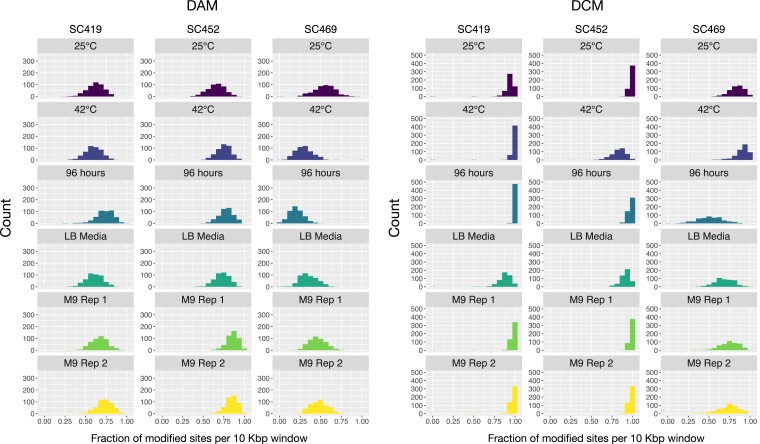
The fraction of DAM 6 mA and DCM 5 mC methylated sites within 10-Kbp windows varies according to strain and growth condition. The histograms in each panel indicate the distribution of 10-Kbp windows in which a certain fraction of sites are DAM (left panel) or DCM (right panel) methylated. This fraction ranges from almost 100% of all sites in all windows (e.g. for SC419 DCM in the 42°C growth condition) to less than 50% of all sites in the majority of windows (e.g. for SC469 DAM in the 42°C growth condition). With the exception of the LB-rich media sample, all cultures were grown in M9 minimal glucose media.

We then used this data to look at large-scale variation in methylation marks across the genome, on the basis of both strain and growth environment. Rather than consider single sites, which exhibit considerable noise in being classified as methylated or unmethylated, we calculated the fraction of methylated sites in 10-Kbp windows across the genome (approximately 500 windows total for a 5 Mbp genome; ‘see *Methods*’). Again, this fraction is indicative of the fraction of sites that tend to be more or less methylated, not a fraction of sites that are (or are not) methylated. Each of these windows contained approximately 40 DAM or DCM sites. We found that the fraction of sites that we classified as methylated within each 10-Kbp window varied by methyltransferase, strain, and environment (Fig. 3), with fewer than 50% of all motif sites classified as methylated in some cases. However, we emphasize that these results do not establish motif sites as modified or not, only less or more methylated. Here we have classified sites as modified based on a threshold to simplify the identification of general large-scale patterns of methylation.

Overall, we inferred that a much higher fraction of DCM sites was methylated compared to DAM sites (Fig. 3). This difference is partly driven by the fact that the signal differences between methylated and unmethylated cytosines at DCM sites are much larger than between methylated and unmethylated adenines at DAM sites (Fig. 2). Thus, it does not reflect biological differences but differences in the sensitivity of each statistical test. Nonetheless, we observed that in some growth conditions, a strain could exhibit similar levels of methylation at DCM and DAM sites (e.g. SC452 at 42°C) whereas another strain in the same condition could exhibit different levels of methylation (e.g. SC469 at 42°C). This indicates that it is unlikely that the lower levels of DAM methylation are due solely to decreased sensitivity, but instead to differences in the activity of each methyltransferase.

We also observed general strain-specific differences in methylation, for example, generally lower levels of both DCM and DAM methylation for SC469. However, it is difficult to determine whether this reflects real differences in methyltransferase activity between strains, or whether it is an artifact of the data analysis: for all cases, we inferred methylation status from a single unmethylated WGA dataset for each strain, and this in itself may cause differences in inferred methylation levels.

**Fig. 4.**
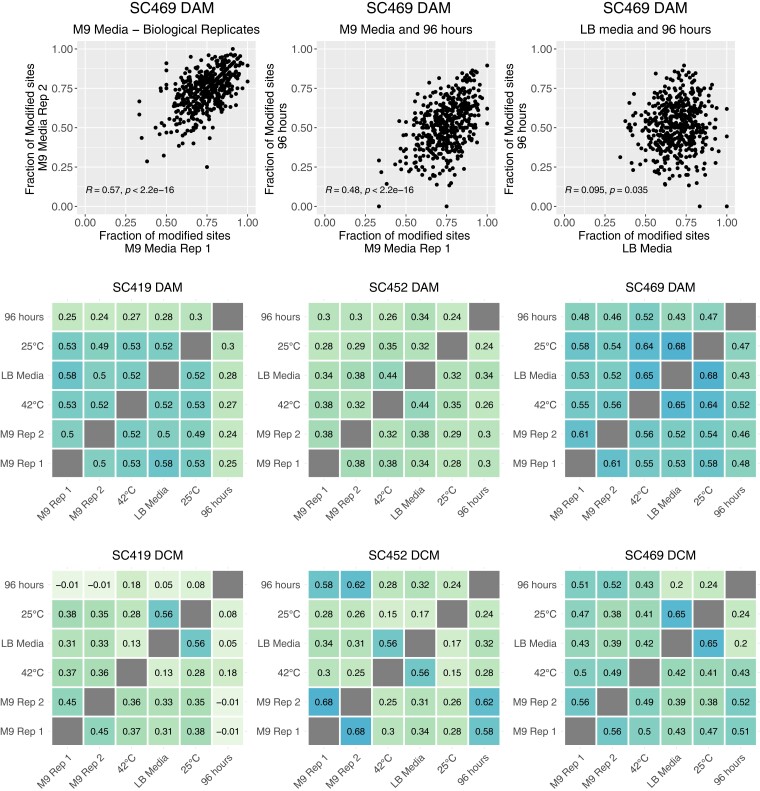
Top row: The fraction of methylated sites in 10-Kbp windows across the genome is correlated across growth conditions. The three panels indicate the fraction of methylated DCM sites within a 10-Kbp window that we inferred as methylated for strain SC469. We observed strong positive correlations in methylation patterns in replicate cultures of minimal M9 glucose media, slightly weaker correlations between M9 media and 96-h stationary phase cultures, and almost no correlation between patterns in rich LB media and 96 h of stationary phase. Pearson partial correlations and corresponding *P*-values are indicated in each plot. Middle and bottom rows, respectively: Pairwise partial correlations in DAM and DCM methylation patterns between all growth environments accounting for genome coverage. Each panel shows all pairwise Pearson partial correlations between growth conditions in the fraction of methylated sites for all 10-Kbp windows in the genome, controlling for genome and WGA coverage in each of the growth conditions.

We next considered whether there were more localized patterns of methylation across the genome. To do this, we tested for correlations in the fraction of methylated sites within the 10-Kbp windows between growth conditions within a single strain. Across different sets of growth conditions, we found that within a strain, some 10-Kbp windows consistently had the majority of sites methylated, while other windows had many fewer sites methylated (Fig. 4). It is likely that some of this is due to differences in coverage, as the relationship between inferred methylation status and coverage was not totally mitigated by our subsampling scheme (‘see *Methods*’). To minimize this dependence, we calculated the partial correlations in methylated fractions for each 10-Kbp window accounting for genome coverage (‘see *Methods*’).

We calculated pairwise correlations in the fraction of methylated sites in 10-Kbp windows across the genome for both DAM and DCM in each strain across all pairs of growth conditions. We found replicable differences across the genome in methylation fractions (Fig. 4), with the correlations between some conditions being higher than others. Critically, we found that in all cases except one the replicate cultures grown in M9 minimal glucose media at 37°C exhibited the strongest correlation with the other M9 replicate. For example, for strain SC469 DCM the partial correlation between M9 replicates 1 and 2 was 0.56. The second strongest correlations for each were with cultures at 96 h extended stationary phase (0.51 and 0.52 for replicates 1 and 2, respectively). Similarly, for SC469 DAM, the correlation between M9 replicates was 0.61. The second strongest correlations for each replicate were with growth at 25°C (replicate 1, 0.58) and growth at 42°C (replicate 2, 0.56).

This pattern, in which each M9 minimal media replicate correlated most strongly with the other replicate, extended to almost all strains and methyltransferases, with the single exception of DAM in strain SC419, for which methylation patterns correlated very similarly for all pairs of conditions (Fig. 4, rightmost panel). As there are a total of six independent growth conditions, there is only a one in five chance that the two M9 replicates are most highly correlated. Thus, the likelihood that they would be the most highly correlated in almost all strains for both DCM and DAM strongly suggests there are growth condition methylation states, despite many pairs of growth conditions being similar. For example, methylation levels for replicate cultures grown in minimal M9 glucose media at 37°C were consistently more similar to each other than others grown in M9 at 42°C.

In addition to high correlations between identical growth conditions, we often found consistent correlations in methylation status between different growth conditions. For example, the methylation patterns in the rich media LB condition (grown at 37°C) often exhibited very strong correlations with methylation patterns in the minimal media 25°C growth condition. In three cases (SC469 DCM, SC469 DAM, and SC419 DCM), these two conditions exhibited the strongest correlation of any pair of conditions. The convergent methylation states in these two conditions may be driven by similar changes in transcriptional activity, which could have an inhibitory effect on methylation.

The lowest levels of correlation we observed were for 96-h extended stationary phase for strain SC419 DCM (Fig. 4, rightmost panel). In some cases, the partial correlations were slightly negative. However, we found that many of the 10-Kbp windows in this condition had almost 100% of all DAM sites methylated (Fig. 3, right panel). Such low variability in methylation status makes it unlikely that strong correlations will occur.

**Fig. 5.**
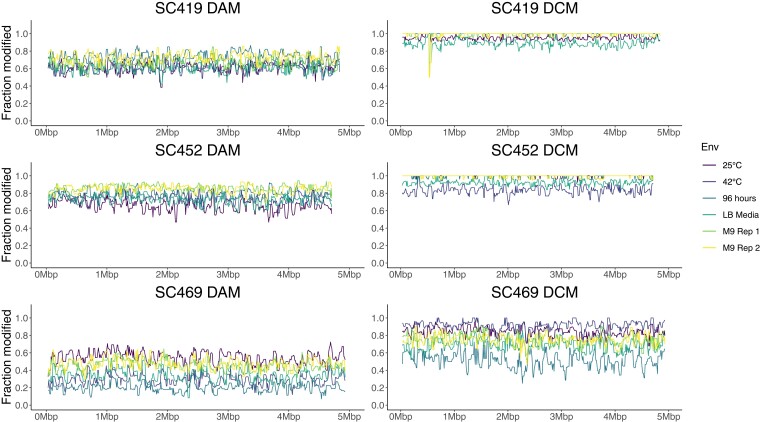
Genome-wide patterns in the fraction of methylated sites. Each panel shows the fraction of methylated sites in 10-Kbp windows across the entire genome, with different growth conditions indicated in different colours. No strong long-range correlations, such as higher methylation at the replication terminus, were apparent (although see main text).

One explanation for the correlations in methylation fractions across growth conditions is that there are consistent long-range intragenomic correlations driven by periodicity in methylation, e.g. methylation fractions are generally lower at the origin of replication and higher at the terminus, or that there is transient methylation behind the replication fork (Anton and Roberts 2021). This would be apparent as long-range correlations in the fraction of methylated sites across the genome. For example, any two windows separated by a distance that is similar to the periodicity should exhibit positive correlations. However, plotting the fraction of methylated sites across the genome revealed no strong long-range patterns (Fig. 5). Careful examination revealed a single case with subtle periodicity, DAM methylation in SC469 (bottom left). In this case, a trough and peak in the fraction of methylated sites occurred precisely at the origin and terminus of replication, respectively (Supplementary Fig. 7). This pattern is unlikely to have arisen from systematic differences in the coverage of native or WGA reads, which we corrected for. In addition, our null expectation is that the region around the origin will have higher coverage during exponential growth. However, we observed that the frequency of modified sites near the origin was lower than elsewhere. This result is the opposite of what we would expect if these observed differences in the fraction of methylated sites were driven by differences in coverage, as higher coverage generally increases the chance of observing modified sites (Supplementary Fig. 1).

To systematically test for long-range correlations in methylation levels, we calculated correlations in the fraction of methylated sites within windows of increasing size, from 250 bp to 500 Kbp, separated by distances of increasing size, from 0 bp to 1 Mbp. This is similar to calculating an autocorrelation function, but for almost all step sizes (‘see *Methods*’), and can reveal patterns that are not easily observed otherwise. Again we found no strong patterns of correlation between any windows larger than 5 Kbp, nor windows separated by a distance of more than 5 Kbp (Supplementary Figs. 8 and 9).This suggests that short-range correlations dominate.

Finally, to test whether these data allow insight into fine-scale methylation patterns, we tested whether there were systematically lower levels of DAM methylation at the region of the *dnaA* locus. This locus is known to remain hemi-methylated for approximately ten minutes following replications due to the binding of the SeqA protein. In minimal media, division occurs approximately every hour (with chromosomal replication on a similar timescale). Thus, this region should be slightly less methylated than other chromosomal regions, although for the majority of the cell cycle, it will have similar levels of methylation (as the period of hemi-methylation is relatively short compared to the timing of replication). Examining this locus in minimal media cultures, we found no strong patterns of lower methylation at the *dnaA* locus (Supplementary Fig. 10). However, this pattern was also absent in LB cultures, which have much more rapid rates of chromosomal replication, and should have a larger fraction of hemi-methylated molecules. These results suggest that this method cannot accurately resolve fine-scale differences in methylation.

## Discussion

Here, we have identified DNA modifications in three *E. coli* natural isolates across a range of growth conditions using ONT sequencing. We have shown that it is possible to determine the motifs at which DNA modifications occur, and that these match the motifs expected given the restriction modification systems present in each genome. However, we also found one motif (CCGG) for which we could not identify a matching RM system; this motif may be modified by a novel methyltransferase.

Furthermore, we have shown that by using a simple binary classification of sites as methylated or unmethylated, it is possible to discern replicable and consistent differences in localized methylation frequency across the genome. While some previous work has suggested that the vast majority of motif sites are methylated during growth (more than 97% (Cohen *et al*. 2016)), other results indicate that a considerably lower fraction of sites are modified, with 60% being estimated as fully modified and 40% partially modified (in this case, it was not possible to distinguish hemi-methylated sites from heterogeneously modified motif sites; (Fang *et al*. 2012)). Our results here are consistent with these latter results although we emphasize that the methylation levels we infer are not indicative of whether a site is or is not methylated, but whether it tends to be more or less methylated than other sites in the genome. We note also that it is necessarily true that during any condition of cell growth, not all motifs are methylated, which again is consistent with our results. There are at least two reasons to expect variability in methylation levels across motif sites. First, there are a number of cases in which methylation levels are known to vary locally due to transcription factors (e.g. several sites within the *pap* operon), or for which methylation excludes transcription factor binding (e.g. at the *agn43* locus). Secondly, while methyltransferases are generally quite processive, and rapidly methylate hemi-methylated sites, at fully unmethylated sites the rate is more than an order of magnitude lower (Hermann *et al*. 2004). Thus, if sites are fully unmethylated, this state may be somewhat persistent.

The methylation patterns we have observed are dependent on growth conditions, with specific localized regions (on the order of thousands of kilobases) in the genome tending to be more or less methylated. These conclusions differ from some previous work. A study on diverse strains of *M*ycobacterium *tuberculosis* showed that most differences in methylation across the genome (as determined via SMRT sequencing) are due to stochasticity in intracellular methylation, rather than consistent differences between cells in methylation rates. Consistent differences between loci in methylation (hypomethylation) were found to be exceedingly rare, on the order of 10–20 sites across the genome (Modlin *et al*. 2020). Methylation levels has also been found to be remarkably consistent across different growth conditions, including antibiotic stress (Cohen *et al*. 2016) and over the growth cycle (Payelleville *et al*. 2018). A significant difference between these latter two studies and the data we present here is the inclusion of methylation at DCM sites (CCWGG) in addition to DAM sites (DAM). Furthermore, these data detect DCM more sensitively than DAM methylation, and the most notable methylation patterns that we find—although subtle—are due to differences at DCM sites (Fig. 4). Differential methylation at DCM sites has been connected to major changes in ribosomal gene regulation (Militello *et al*. 2012). Notably, some taxa exhibit clear increases in methylation from the origin of replication to the terminus (Seong, Roux, *et al*. 2021; diCenzo *et al*. 2022), which we also found evidence of (Supplementary Fig. 7).

Critical to our proposal that DNA methylation patterns have epigenetic effects is that methylation is heritable. Sites at which both the top and bottom strand are methylated will impart hemi-methylated strands to both daughter cells, which will become fully methylated by “maintenance” methyltransferases (Anton and Roberts 2021); sites that are hemi-methylated will impart one hemi-methylated strand to one daughter cell and one unmethylated strand, which is more likely to remain unmethylated. This means that mother cells with methylation at a certain genomic location will have daughter cells that are also methylated at that location, but this will vary across daughter cells. Thus, if methylation affects phenotype, and methylation varies between individual cells in a population, then it acts as an epigenetic mark for the instances we have described here.

It is possible that there are unrecognized causes that drive some of the inferred differences in methylation status across the genome. For example, subtle differences in nucleotide context affect both the activity of the methyltransferase and the deviations in ONT signal. This undoubtedly influences our ability to accurately infer methylation status. However, we do not expect these differences to be dependent on growth conditions. Thus, the fact that we find both higher correlations between identical growth conditions, and consistently higher correlations between specific pairs of growth conditions [e.g. rich media (LB) at 37°C and M9 minimal glucose media at 25°C], suggest that nucleotide context is not the only force driving this correlation in methylation states. Additional work is required to test the repeatability of methylation patterns in different conditions, and whether other divergent growth conditions, for example, antibiotic stresses or additional heat stress, lead to greater differences in methylation patterns. Similarly, methylation patterns should converge as growth conditions converge—for example—we would expect more similar patterns comparing methylation during growth at 37°C and 39°C than to 42°C.

In eukaryotes, it is well-established that methylation affects gene expression (Song *et al*. 2005; Vanderkraats *et al*. 2013), and thus cell phenotypes. Here we have shown that methylation patterns are consistent and replicable in different growth conditions in *E. coli.* In addition, for identical growth conditions (in the data here, M9 minimal glucose media), there are strong correlations in which specific regions of the genome are methylated. There are two readily apparent explanations for these results. Either growth phenotypes affect patterns of methylation, or methylation patterns affect growth phenotypes (or both). We propose that it is likely that (as with eukaryotic cells) methylation affects gene expression in *E. coli* in different growth environments, although we have not established causation. This connection between methylation and transcriptional regulation has been proposed previously (Beaulaurier *et al*. 2015), and there are data that both support (Gaultney *et al*. 2020) and refute the hypothesis that methylation always affects gene expression (Mehershahi and Chen 2021). However, we note that there are a large number of other well-established instances in which this causal direction has been established (Sánchez-Romero and Casadesús 2020).

Regardless of whether methylation functions as an epigenetic mark, and regardless of its causality, we have shown that just as bacterial cells undergo transient differentiation into different growth phenotypes, they also undergo transient differentiation into distinct methylation states. As we have not used synchronized cultures, it is unlikely that the correlated methylation is due to synchrony in the cell cycle that differs between growth conditions. This is further supported by the fact that we have shown that correlations do not arise because of short- or long-range correlation in methylation fractions, although we find subtle indications of higher methylation at the terminus in one case (Supplementary Fig. 7c). Rather, we hypothesize that these correlations arise from localized differences across the chromosome that are maintained during growth under specific conditions.

This work raises the possibility of discerning bacterial growth states without measuring cell physiology or quantifying the transcriptome, similar to what can be done for differentiated eukaryotic cells. We propose that with sufficiently long reads and precise measurements, it will be possible to quantify methylation states across single molecules, and from there infer the growth state of a cell from which a particular DNA molecule has originated. In addition, with more nuanced model-based or machine-learning analyses, it may be possible to assign genomic methylation patterns more specifically to specific growth states. This contrasts with more standard approaches such as single-cell transcriptome profiling, which is often of limited use in bacteria given the extremely small number of transcripts contained in most cells.

## Supplementary Material

jkac310_Supplementary_Data

## Data Availability

The files generated by Nanodisco specifying each genome position and the *P*-values from tests of differences in the signal of the whole-genome amplified DNA and native DNA are available as RDS files from Figshare at https://doi.org/10.6084/m9.figshare.21569292. The original fast5 files are available through ENA Accession PRJEB57097. Analysis files are available at https://github.com/GeorgiaBreckell/Methylation. Supplementary figures are available on Figshare at https://doi.org/10.6084/m9.figshare.21569292.

## Notes

*Communicating editor: B. Andrews*
