# Supplementary material for: Growth condition-dependent differences in methylation imply transiently differentiated DNA methylation states in Escherichia coli
Source: G3 (Bethesda). 2022 Dec 1;13(2):jkac310. doi: 10.1093/g3journal/jkac310 (PMC9911048; doi:10.1093/g3journal/jkac310)
Supplement: jkac310_Supplementary_Data [file jkac310_supplementary_data.pdf]

# Supplementary Figures

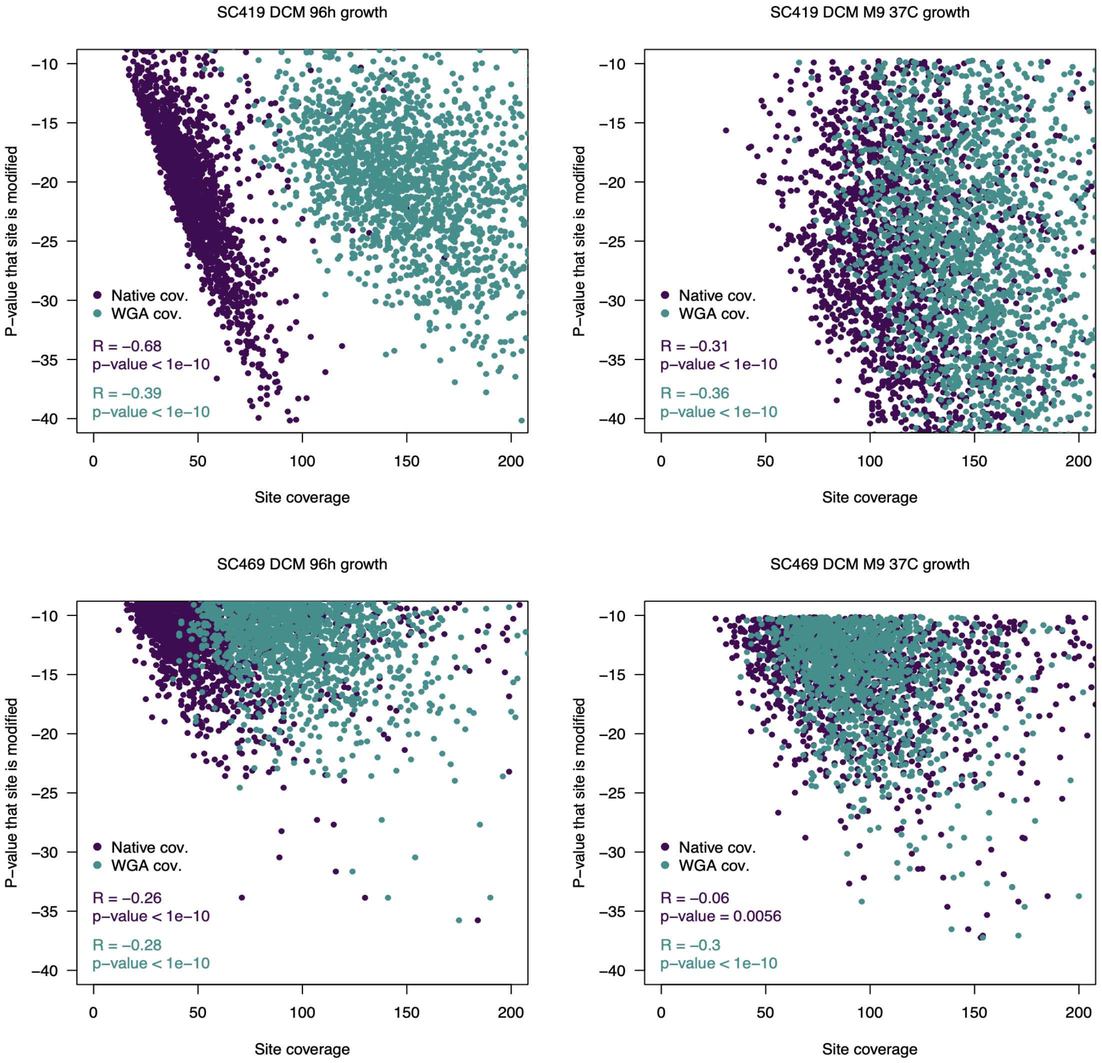


**Figure S1. Correlation between coverage and the Nanodisco-derived p-values.** Each point indicates the coverage at individual DAM or DCM sites and the p-value of the Nanodisco Mann-Whitney U-test.. There is a clear relationship between the likelihood the p-value returned by Nanodisco (indicating a site is likely modified) and the coverage at that site, with both the coverage of the native DNA sample and the WGA sample affecting the test implemented by Nanodisco. The four examples above are all for DCM sites in two strains and two growth conditions for each. In all plots, only the sites that have p-values significantly lower than the null model background are shown. The native coverage at these sites is shown in purple; the WGA coverage at these same sites is in blue. For both native and WGA coverage, there is a strong negative correlation - sites with higher coverage have a lower p-value and a higher probability of being identified as methylated, although this differs between datasets. For example, there is only a weak relationship (R = -0.06) between native coverage and the p-value to the test in the SC469 DCM dataset.

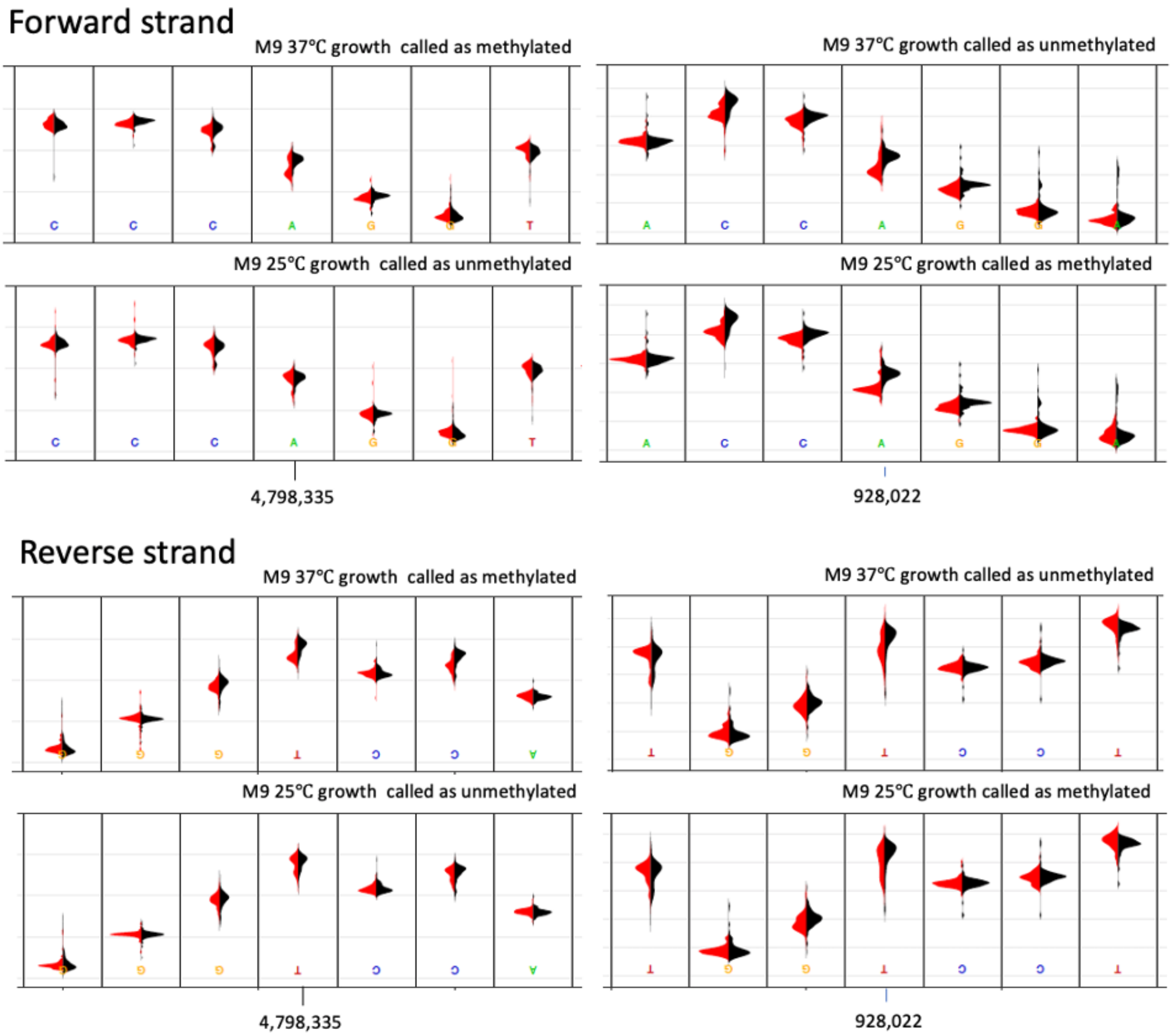


**Figure S2. Raw nanopore signal distributions on the forward and reverse strands at identical genomic locations of DCM sites that we inferred as methylated (top panels in each pair) or unmethylated (bottom panels in each pair).** The change in the DCM CCwGG methylation status is apparent as a shift in the distribution of the red curves at the A / T position outlined with the blue box. In black are reads from the control (unmethylated whole genome amplified DNA); in red are the native DNA signals. In many cases, the shift in signal is subtle. However, the identification of these sites as methylated or unmethylated is a binary classification of a continuous state - sites that we identify as unmethylated may in fact be methylated in 40% of all cells; sites we identify as methylated may be methylated in only 60% of all cells.

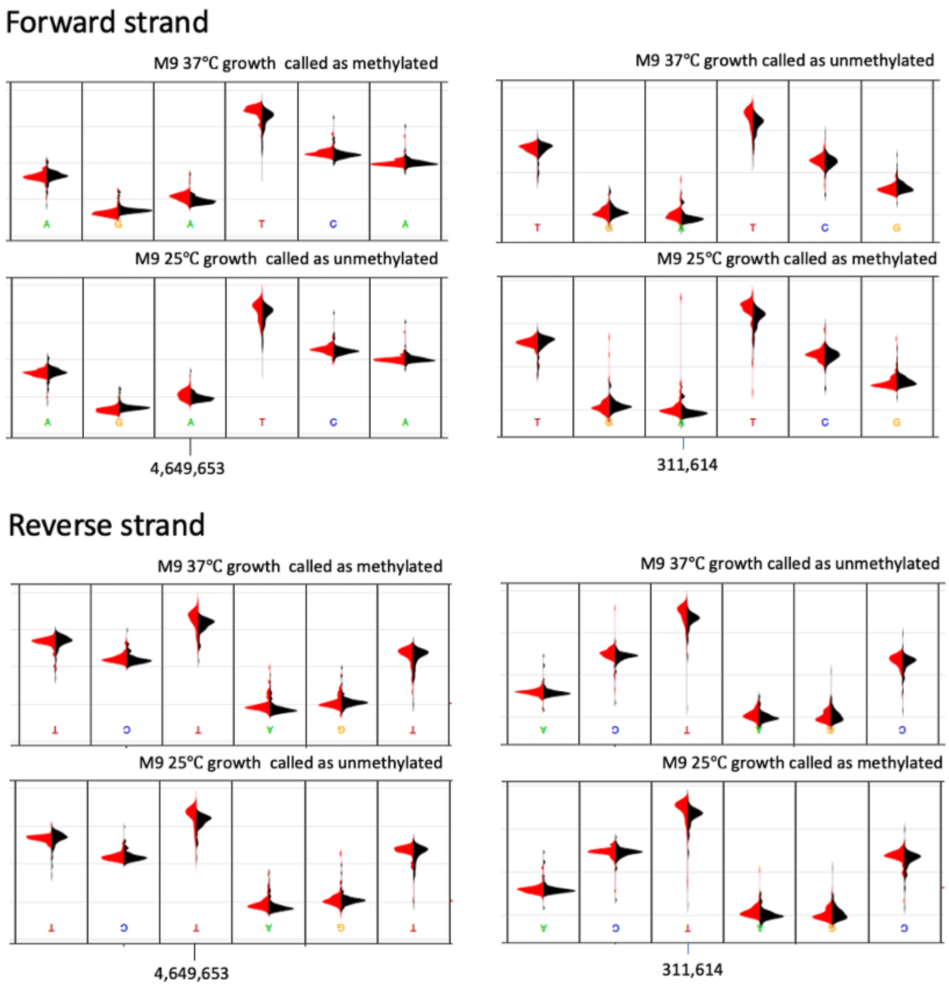


**Figure S3. Identical DAM sites are inferred as methylated or unmethylated across different growth conditions.** The change in the DAM GATC methylation status is apparent as a shift in the distribution of the raw nanopore signal from native DNA (red curves) at the T and A positions (the A is the modified base) compared to WGA unmodified DNA (black curves). Left panels: a DAM 6mA site that we inferred as methylated in M9 37°C growth (top) but not during 25°C growth (bottom). This is most apparent as a shift in the signal at the T position, for which the overlap between red and black is less in the top panel. Right panels: a DAM GATC site that we inferred as unmethylated in M9 37°C growth (top) but methylated during 25°C growth. Again, this is most apparent as a shift in the signal at the T position, with the overlap being higher in the top panel. Note that all native DNA molecules are not necessarily methylated at positions that we call as methylated, and vice versa: at positions that we call as unmethylated, all molecules are not necessarily unmethylated.

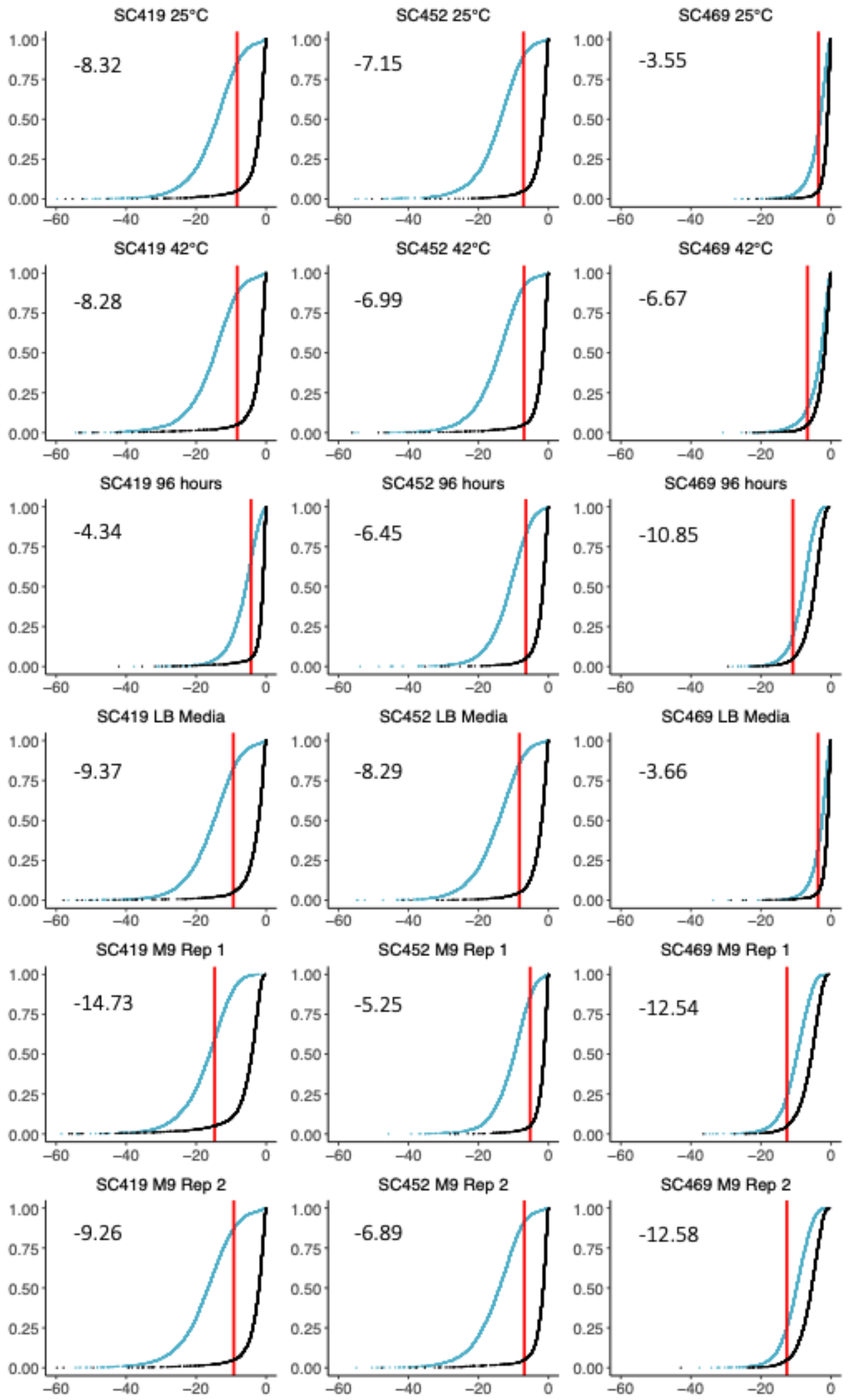


**Figure S4. Cumulative distributions of p-values for DAM sites relative to random (unmethylated) sites.** For each combination of isolate and growth condition we used the distribution of p-values at DAM binding sites (blue) and an equal number of random sites (black) to determine a p-value cutoff. This cutoff was established such that 10% of all unmodified sites were inferred as being modified, equivalent to a 0.1 FDR. Each cutoff is shown in red, and the log10 of the p-value cutoff is noted within each plot.

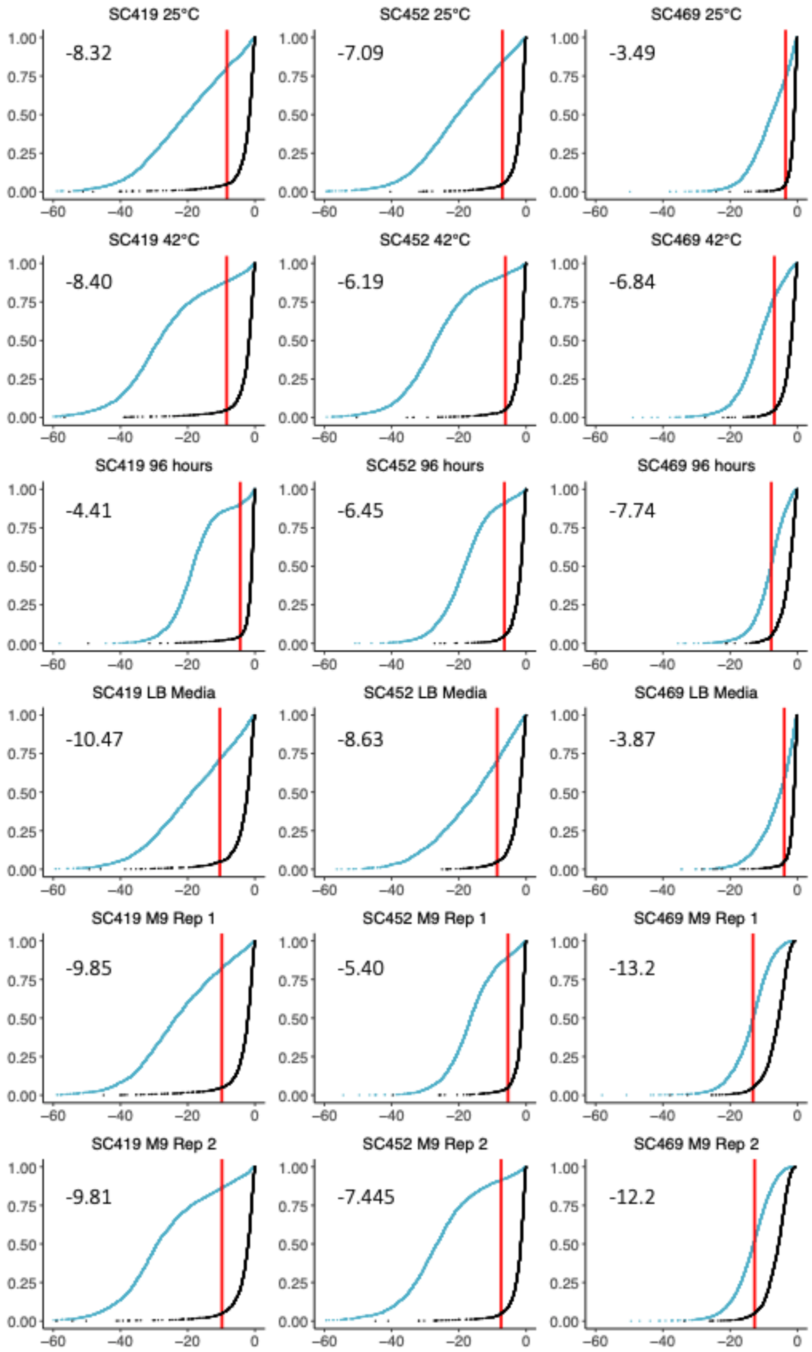


**Figure S5. Cumulative distribution of p-values for DCM sites relative to random sites.** For each combination of isolate and growth condition we used the cumulative distribution of p-values at DCM binding sites (blue) and an equal number of random sites (black) to determine a p-value cutoff equivalent to an FDR of 0.1. Each cutoff is shown in red, and the log 10 of the p-value cutoff is noted within each plot.

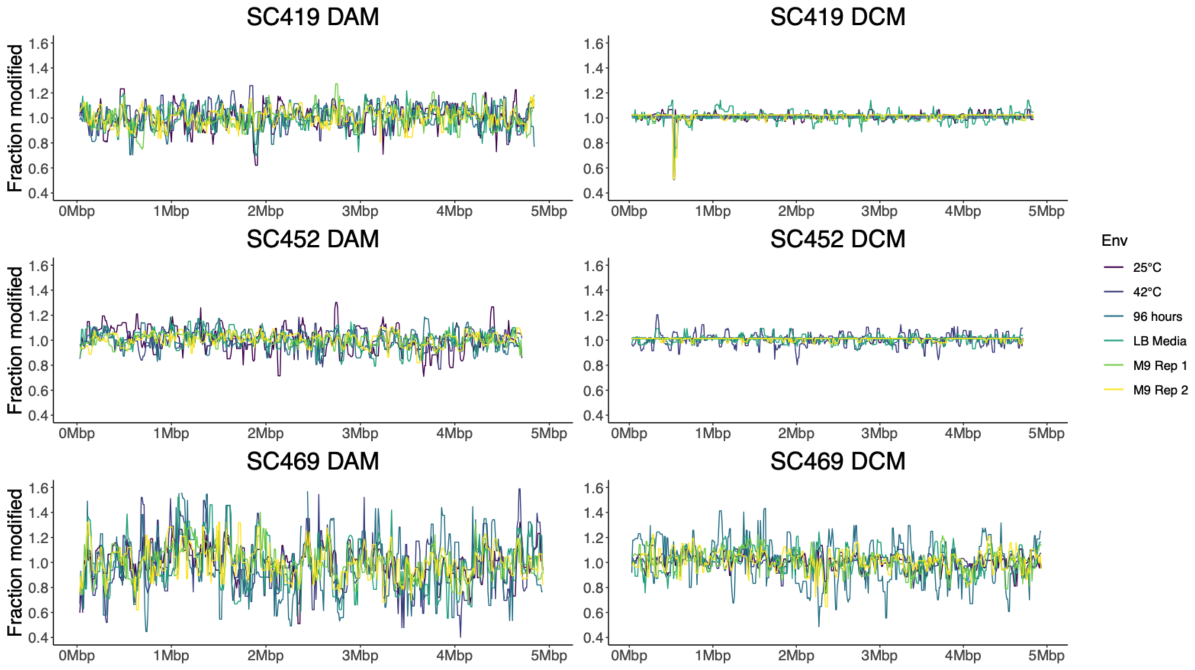


**Figure S6. Mean-normalised fractions of modified sites across the genome.** For each growth condition, we divided the fraction of modified sites in each window by the mean fraction of modified sites across all windows for that growth condition. This normalised fraction of modified sites are generally consistent across the genome for each methyltransferase and strain, which is clearly apparent in Fig. 4.

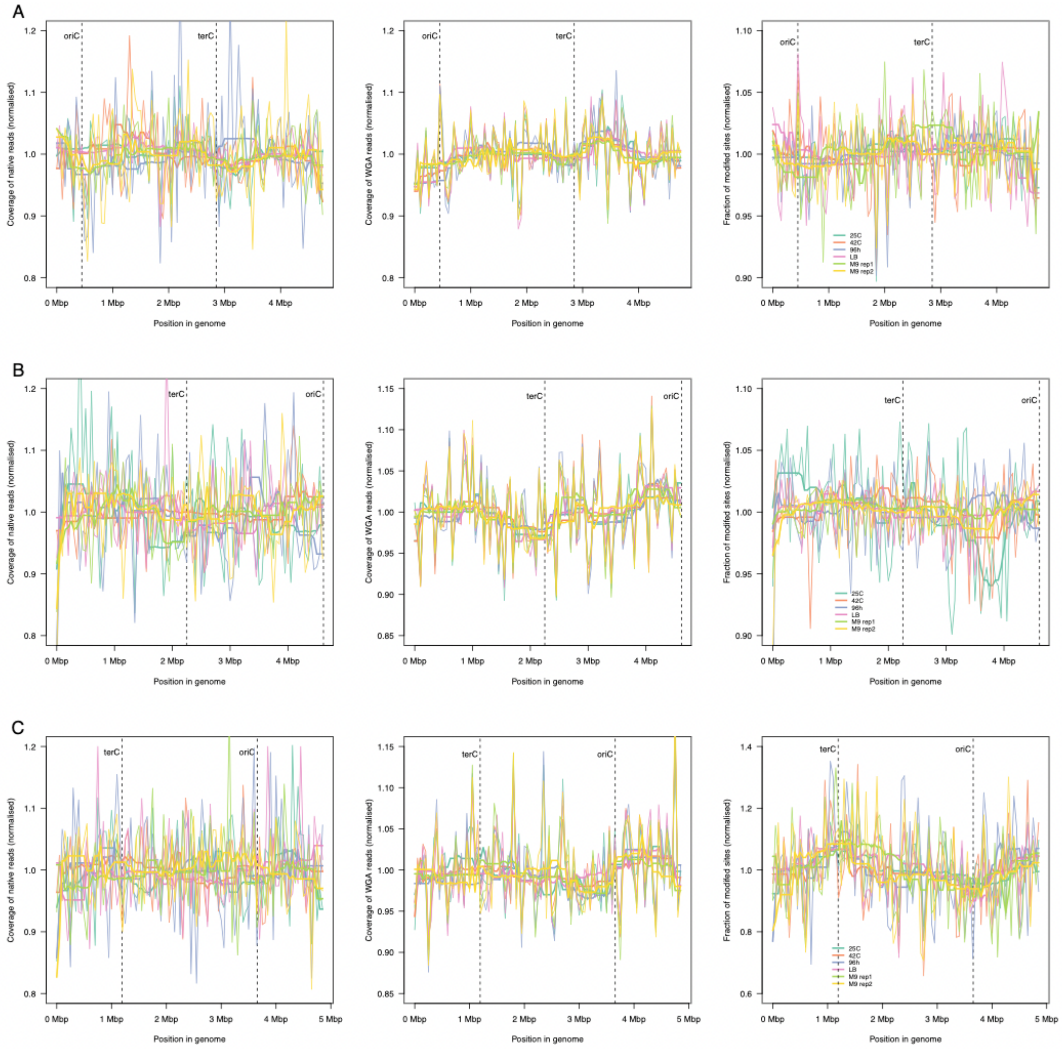


**Figure S7. Mean-normalised read coverage and fractions of DAM-modified sites across the SC469 genome.** (A) Strain SC419; (B) Strain SC452; (C) Strain SC469. Each panel shows, from left to right: coverage of native reads; coverage of whole genome amplified reads; the fraction of sites inferred as modified. The DAM modification data here are the same as shown in Figure S6 with a larger smoothing window (50 Kbp) to emphasise large scale variation in modifications. Running medians for each growth condition are shown in thicker lines (running window over 600 Kbp). The locations of the *oriC* and *terC* sites are indicated with vertical dotted lines. The smoothed data in the right-most panel in (C) clearly indicate that the *oriC* and *terC* regions of SC469 are associated with a trough and peak in the fraction of methylated sites, respectively despite there being no consistent decrease or increase in WGA or native coverage in those regions. Note that although only a single WGA dataset was used, different subsamples were taken, so coverage is not identical across all conditions (centre column).

45

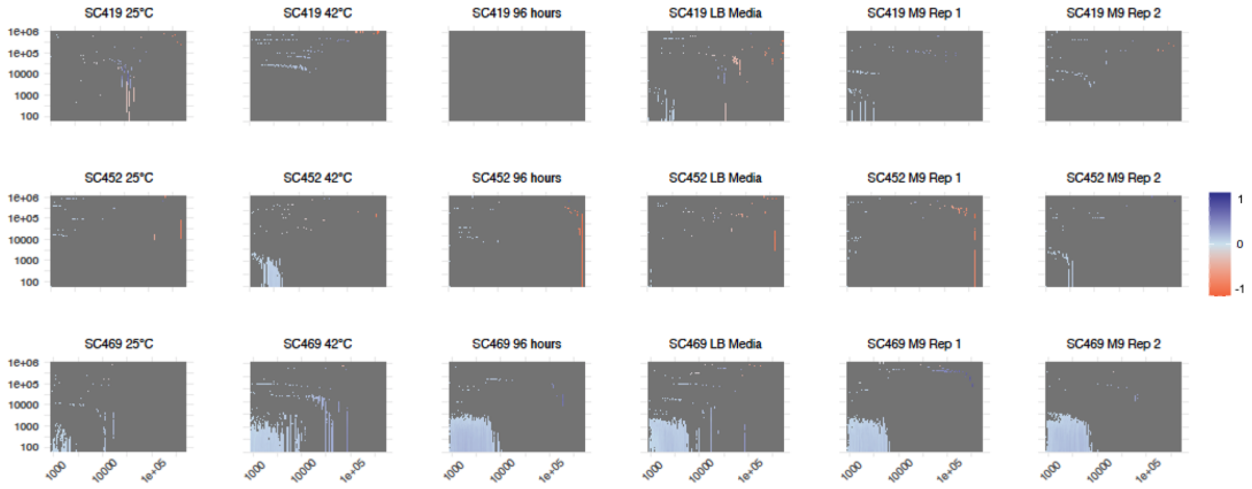


**Figure S8. Global autocorrelation plots for DCM methylation**. Each panel is a heatmap showing the correlation for the fraction of methylated DCM sites between windows of increasing size, ranging from 250 bp to 500 Kbp (different window sizes are plotted in columns), separated by increasing distances ranging from 0 (i.e. adjacent windows) to 1 Mbp (different distances are plotted in rows). Window sizes increase by a constant fraction of 4.7%; separating distances increase by a constant fraction of 9.6%. For example, the bottom left square in each heatmap shows the correlation in the fraction of methylated sites for neighbouring 250 bp windows; the middle square in each plot shows the correlation between 20.9 Kbp windows separated by 7.6 Kbp; the top right indicates 500 Kbp windows separated by 1 Mbp. In the example here, a standard autocorrelation function (ACF) would plot the correlations between windows of a certain size separated by a specific number of windows (e.g. 10 Kbp windows separated by 0 bp (neighbouring), 10 Kbp (one window), 20 Kbp (two windows), etc. This would be similar to several squares in the 53rd column in this plot: the squares in rows 1 (0 bp distance between windows), 56 (10 Kbp distance), 64 (20 Kbp distance), 68 (30.2 Kbp distance), 71, 74, and 76. However, this plot shows the analogous set of correlations at almost all window sizes and distances. For clarity only correlations with $p < 0.01$ are shown. In almost all cases, the correlations are positive (i.e. windows that are close tend to have similar levels of methylation), but this correlation only exists for windows up to approximately 5-8 Kbp in size and separated by a maximum of 5 Kbp. This suggests that there are no long range correlations in the fraction of methylated sites. Note that the strongest correlations are observed for strain SC469, which is also the strain that exhibited the greatest variance in fraction methylated across genomic windows (**Fig. 3**). For other strains, the low level of variance in methylated fractions necessarily weakens the correlations.

46

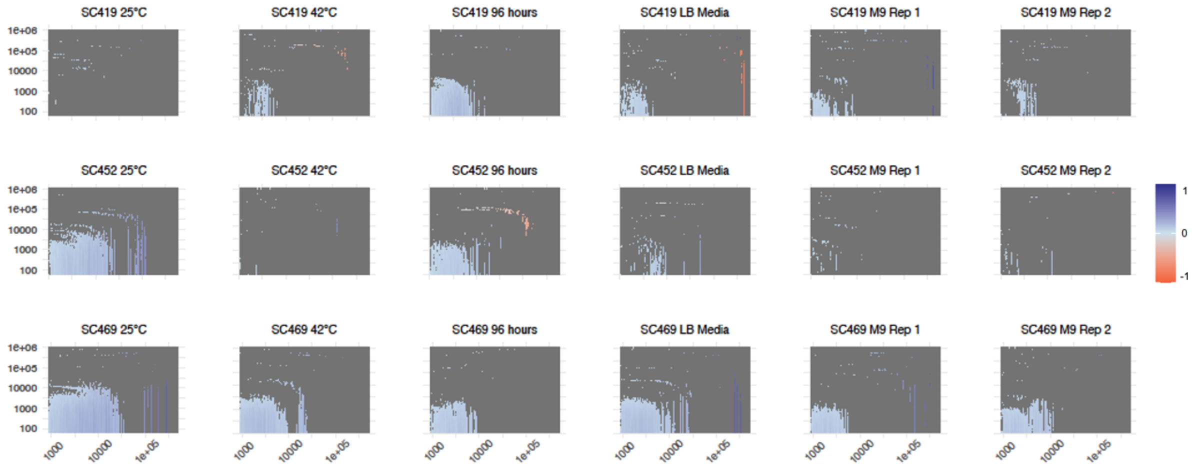


**Figure S9. Global autocorrelation plots for DAM methylation.** The annotation and details of this plot are the same as those shown in **Fig. S9** but for DAM methylation. Again, for clarity only correlations in $p < 0.01$ are shown. The correlations here in the fraction of methylated sites in a window are in general stronger, but extend to a similar distance to those observed for DCM. Again, The strongest correlations are observed for strain SC469. However, correlations are also apparent for other strains in other conditions, also most likely due to the fact that DAM methylated fractions exhibited much greater variation than DCM (**Fig. 3**).

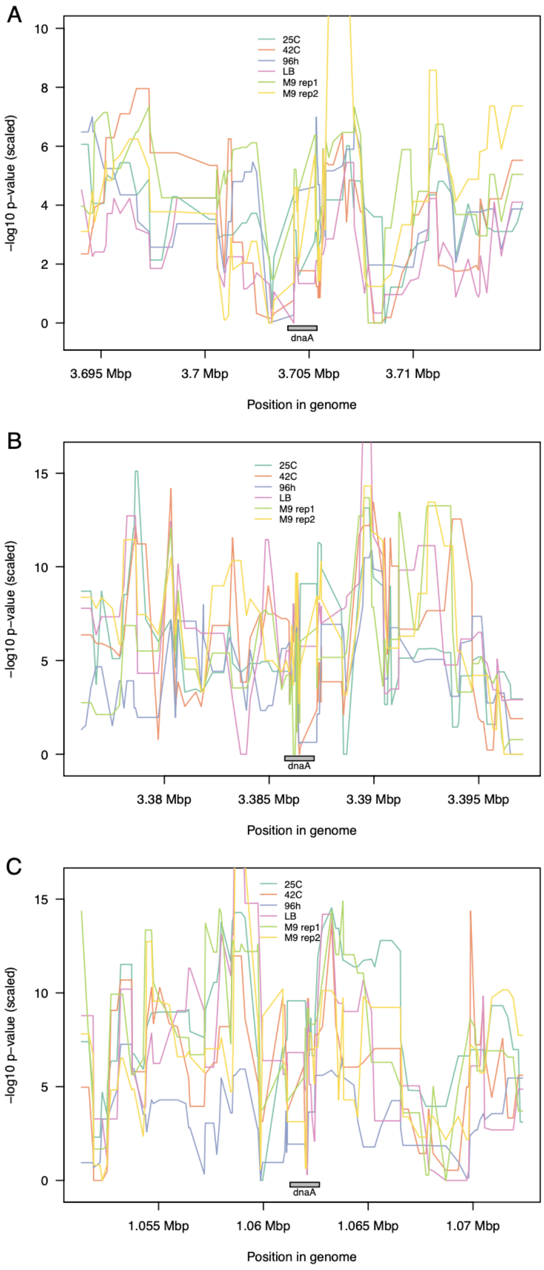


**Figure S10. Methylation profiles at DAM sites proximal to *dnaA*.** Each panel indicates the pattern of methylation in a 20 Kbp window surrounding the *dnaA* open reading frame. For each DAM site, a p-value is indicated on a -log10 scale. The p-values are correlated with the likelihood that a site is methylated, with lower p-values (higher -log10 p-values) associated with sites that tend to be more methylated. To decrease variability, the p-values shown are calculated as a running median over five DAM sites. To facilitate comparisons between samples and minimise artifactual differences in p-values, for example arising from differences in data quality, the minimum p-value has been subtracted from all sites across a sample, such that each sample has a minimum -log10 p-value of 0. We expect that at the *dnaA* locus, sites will be hemi-methylated for some time after replication due to *seqA* binding. There is little evidence to indicate this, suggesting that the necessary resolution is not present in this dataset. (A) SC469; (B) SC452; (C) SC419.
